# Role of Epigenetics in Chronic Lung Disease

**DOI:** 10.3390/cells14040251

**Published:** 2025-02-10

**Authors:** Felix Ritzmann, Michelle Brand, Robert Bals, Michael Wegmann, Christoph Beisswenger

**Affiliations:** 1 Department of Internal Medicine V—Pulmonology, Allergology and Respiratory Critical Care Medicine, Saarland University, 66421 Homburg, Germany; felix.ritzmann@uks.eu (F.R.); michelle.brand@uks.eu (M.B.); robert.bals@uks.eu (R.B.); 2 Helmholtz Institute for Pharmaceutical Research, 66123 Saarbrücken, Germany; 3 Division of Lung Immunology, Priority Area Asthma and Allergy, Research Center Borstel—Leibniz Lung Center, 23845 Borstel, Germany; mwegmann@fz-borstel.de; 4 Airway Research Center North (ARCN), German Center for Lung Research (DZL), 23845 Borstel, Germany

**Keywords:** epigenetics, lung disease, trained immunity

## Abstract

Epigenetics regulates gene expression and thus cellular processes that underlie the pathogenesis of chronic lung diseases such as chronic obstructive pulmonary disease (COPD), asthma, and idiopathic pulmonary fibrosis (IPF). Environmental factors (e.g., air pollution, smoking, infections, poverty), but also conditions such as gastroesophageal reflux, induce epigenetic changes long before lung disease is diagnosed. Therefore, epigenetic signatures have the potential to serve as biomarkers that can be used to identify younger patients who are at risk for premature loss of lung function or diseases such as IPF. Epigenetic analyses also contribute to a better understanding of chronic lung disease. This can be used directly to improve therapies, as well as for the development of innovative drugs. Here, we highlight the role of epigenetics in the development and progression of chronic lung disease, with a focus on DNA methylation.

## 1. Introduction

Chronic lung diseases such as chronic obstructive pulmonary disease (COPD), bronchial asthma and idiopathic pulmonary fibrosis (IPF) are characterized by a loss of lung structure and function. In obstructive lung disease, pathological narrowing of the airways occurs due to airway remodelling, which includes disruption of the extracellular matrix, goblet cell metaplasia, mucus overproduction, bronchial wall thickening, and smooth muscle cell proliferation in the airway. In addition, especially in COPD, loss of the lung parenchyma leads to pulmonary emphysema and thus to a massive loss of gas exchange surface [1,2]. IPF is characterized by scarring of the lung parenchyma [3].

Epigenetic modifications, without altering the gene sequence, regulate chromatin structure and thus gene expression [4,5]. This regulation occurs primarily through DNA methylation and histone modifications like acetylation, methylation, and phosphorylation. DNA is covalently methylated by DNA methyltransferases (DNMTs) at the 5’ position of cytosine in cytosine-guanine dinucleotide (CpGs) islands, thereby restricting the binding of transcription factors and regulatory proteins to the DNA and resulting in stable silencing of gene expression [4]. The attachment of acetyl groups by histone acetyltransferases (HATs) to the normally positively charged lysine residues of histone proteins results in an open chromatin structure and makes DNA more accessible to transcription factors and RNA polymerases. Deacetylation has the opposite effect and is mediated by histone deacetylases (HDACs) [5]. The molecular mechanisms underlying epigenetics are discussed in excellent reviews [6,7,8,9].

**Figure 1:**
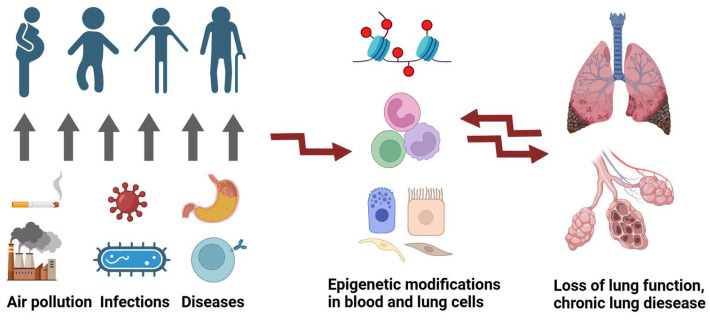
Epigenetic changes occur before a diagnosis is made. Environmental factors (e.g., smoking during pregnancy, infections in early childhood) and diseases (e.g., gastroesophageal reflux) may cause epigenetic changes that increase the risk of loss of lung function and chronic lung disease (Created in BioRender. Beissswenger, C. (2025) https://BioRender.com/o80m047 (accessed on 24 December 2024)).

The epigenome is important during development, as well as in adult organisms; for example, it is important in the maintenance of stem cells and their differentiation [10]. In addition, various studies have shown that the environment interacts with the genome through epigenetic changes [11]. This leads to the altered expression of numerous genes involved in cellular signalling pathways and mechanisms, including those underlying the pathogenesis of chronic lung diseases. Smoking is certainly an external factor that modifies the epigenome in various organs such as the blood or the lungs [12]. However, epigenetic changes that promote chronic lung disease may occur long before a diagnosis is made. Smoking during pregnancy or infections in early childhood may cause epigenetic changes that pass from cell to cell, significantly increasing the risk of chronic lung diseases (e.g., asthma, COPD) later in life (Figure 1) [11].

Here we highlight the role of epigenetics in chronic lung disease, with a focus on DNA methylation. 

## 2. COPD

COPD is mostly caused by cigarette smoke (CS) and is characterized by ongoing inflammation of the lung associated with acute and chronic infections. The main symptoms are airway limitation, loss of lung function, and emphysema [2]. However, a significant proportion of COPD patients have never smoked. Even if self-inflicted damage to the lungs caused by smoking, together with various gene variants, is assumed to be the cause of COPD, COPD is currently understood to be the end result of gene–environment interactions that have accumulated over patients’ entire lifetimes [11,12]. These gene-environment interactions probably begin in utero in smoking mothers and extend through passive smoking and infections in childhood, to smoking, poor nutrition, and alcohol abuse in adolescence and adulthood [11,13]. Current research is therefore starting to address the question of the extent to which such environmental influences lead to epigenetic changes in DNA that contribute to the early loss of lung function and promote the occurrence of COPD in those at risk [11,12,14]. 

Many studies have investigated whether smoking and COPD are associated with epigenetic changes in the DNA of different cells in the bloodstream and lungs and whether epigenetic changes contribute to disease progression. Of particular interest is whether and how epigenetic changes affect the expression of genes that regulate cellular signalling pathways and mechanisms and thus contribute to the different endotypes of COPD and disease mechanisms [12,15]. 

Blood cells are particularly well suited for epigenetic analyses due to their easy accessibility and low variability. Therefore, a number of studies have examined DNA methylation in blood cells from smokers and COPD patients. In a genome-wide analysis, Morrow et al. found multiple methylation sites (e.g., PIK3CD sites with a positive correlation between gene expression and DNA methylation at cg03971555 and cg12033075) that predict all-cause mortality in smokers with and without COPD, regardless of decline of lung function or emphysema progression [16]. Tsaprouni et al. showed in a cohort of European descent that methylation is reduced at numerous genomic loci in DNA from the whole blood of smokers but also found that this is partially reversible after smoking cessation [17]. A widespread effect of smoking on DNA methylation has also been demonstrated in other studies with European and Asian cohorts [18,19,20]. A comprehensive meta-analysis of genome-wide DNA methylation using blood-derived DNA samples from 16 cohorts revealed statistically significant differential methylation of CpGs annotated to 1405 genes. Here, too, it was shown that the amount of methylation at many, but not all, differentially methylated CpG sites returned to that seen in non-smokers five years after individuals quit smoking. A search for these genes in the National Human Genome Research Institute-EBI GWAS Catalog also showed that many of these genes are associated with cardiovascular diseases [21]. In a recent study, Saint-André and colleagues showed that epigenetic mechanisms mediate the persistent effect of smoking on adaptive immune functions of blood cells and that the effects on T cells persisted for years after the individuals smoked [22]. The authors showed that smoking is associated with the production of cytokines in cells of the adaptive immune system via DNA methylation at specific signal trans-activators and regulators of metabolism [22]. 

Overall, the results of the various studies on smoking and methylation are very variable. Smoking appears to lead to numerous epigenetic changes, many of which disappear after smoking cessation, but some of which are still present after many years [23,24]. Most clearly, differential methylation of the aryl-hydrocarbon receptor repressor (*AHRR*) has been linked to smoking in various studies. A longitudinal study showed that methylation at the cg05575921 site in the *AHRR* gene is reversed after smoking cessation [25]. Further studies are needed to clarify whether methylation of *AHRR* and other genes is related to clinical parameters and can serve as a biomarker, e.g., for smoking cessation [15,17,18,22,26,27,28,29].

Beyond smoking, various studies have examined the extent to which DNA methylation of blood cells is associated with and influences lung function in COPD patients and could potentially serve as a biomarker [12,30]. One of the first such studies demonstrated a correlation between DNA methylation at CpG sites and the presence and severity of COPD. This is linked to a number of genes involved in various signalling pathways that regulate inflammation, stress responses, and wound healing [31]. In a single-cohort epigenome-wide association study, 28 CpG sites were discovered in the peripheral blood, 26 of which were associated with lung function and three of which were associated with COPD. The differentially methylated sites mapped to genes involved in cellular pathways including alternative splicing and JAK-STAT signalling [32]. A multiancestry epigenome-wide association meta-analysis revealed 1267 CpGs (1042 genes) that were differentially methylated in relation to lung function and pathways (e.g., Wnt signalling) relevant to pulmonary function. A total of 73 of the identified CpG sites were associated with COPD. By integrative epigenomic analysis and druggable-target analysis, the authors also discovered genes that are targets of investigational or approved drugs, such as is *TNFRSF4* [33]. However, in a study with 1561 subjects, no significant genome-wide association was found between COPD and DNA methylation in never and current smokers [34]. In a systematic review, Casas-Recasens et al. systematically searched the literature and extracted and re-analysed data only from publications that met specified criteria [12]. For example, the studies had to use whole-genome data, had to be related to lung function or COPD, and were not allowed to focus only on one specific cell type. Despite high heterogeneity making a formal meta-analysis impossible, there were consistencies among some of the 10 studies that examined blood cells. In at least two studies, 12 DMPs (differentially methylated probes) and 14 genes were found to be associated with COPD with p-values less than 1 × 10^−4^. The ontologies associated with these genes include erythrocyte differentiation and inorganic anion transmembrane transport. Additionally, 51 DMPs and 69 genes were associated with FEV1 in two or more studies. The ontologies here included immune response, the regulation of CD4 and T-helper cell activation, and cytokine production. Furthermore, 11 DMPs and 12 genes were associated with the FEV1/FVC ratio, and 4 genes (*GPR15*, *AHRR*, *F2RL3*, *GFI1*) were associated with FEV1 % predicted and the FEV1/FVC ratio. However, only *AHRR* was associated with spirometric parameters and COPD [32,33]. In addition, methylation of *RUNX3*, which is linked to lung development, was found to be associated with temporal changes in FEV1 and FVC in the two studies that analysed changes in lung function over time [12,26,27]. 

Epigenetic analyses of lung samples indicate a correlation between smoking and COPD and alterations in methylation patterns in lung cells. Due to the usually small number of samples from the lung, which are more difficult to obtain than blood samples, as well as to their cellular heterogeneity and the different methods used, the studies with tissues or cells from the lung stand on their own. The analysis of brush biopsies showed that smoking is associated with epigenetic alterations and corresponding modifications in gene expression in the small airways [35]. The majority of observed changes in DNA methylation were characterized by a reduction in methylation levels. The top 25 hypomethylated genes included seven genes involved in xenobiotic metabolism, six involved in xenobiotic metabolism signalling and five involved in aryl hydrocarbon receptor signalling [35]. Analysis of bronchial brushing of small airways also showed many DMPs (1260 DMPs [36], 2744 DMPs [37]) associated with COPD that may be related to gene expression and signalling pathways relevant to COPD (e.g., oxidative stress responses). Comparative analysis of parenchymal lung tissue samples from subjects with COPD (all former smokers) and samples from subjects with normal lung function revealed 2456 differentially methylated sites associated with COPD (FDR < 0.05) [38]. Another study with lung tissue from a small number of non-smokers, current smokers, and COPD patients identified 280 CpG sites that were differentially methylated between COPD and non-smokers and 10 CpG sites that were differentially methylated between COPD and smokers (*p* < 0.001) [39]. In addition, a study by Casas-Recasens suggests that the lung methylome is related to the severity of airflow limitation [40]. In that study, 139 differentially methylated positions (78 genes) were identified between COPD patients (former smokers) and never smokers with normal spirometry; additionally, 36 DMPs (13 genes) were associated with mild to moderate COPD (GOLD 1/2) and 99 DMPs (56 genes) were associated with severe COPD (GOLD 3/4, *p* < 10^−8^) [40]. Ringh et al. showed that smoking affects methylation in bronchoalveolar lavage (BAL) cells, with 1756 DMPs associated with smoking. The associated changes in the transcriptome may be related to signalling, migration, and inflammatory responses of immune cells [41]. Ström et al. analysed BAL cells (about 90% macrophages) and found 1155 DMPs between subjects with COPD and those with normal lung function [42]. In a methodologically oriented study with a small number of participants, DMPs were identified that mapped to leukocyte antigen (HLA) genes [43]. COPD also appears to be associated with epigenetic changes in fibroblasts [44]. In a recent study, Schwartz et al. generated genome-wide DNA-methylation maps for lung fibroblasts across COPD stages from a total of 11 individuals [45]. The authors found that in COPD, regulatory regions were affected by DNA methylation and that the genes involved in DMPs and associated with proliferation, DNA repair, and extracellular matrix organization were dysregulated [45]. Overall, a common feature of the lung-tissue studies is the observation that a large number of sites are hypermethylated in COPD, while others are hypomethylated. However, due to the heterogeneity of the studies and the small number of samples, future studies will need to determine which signalling pathways are deregulated by DNA methylation in COPD in a cell-type-specific manner.

In conclusion, it is becoming increasingly clear that a variety of cellular processes and signalling pathways in blood cells, inflammatory cells, and structural cells are dysregulated as a result of epigenetic modifications in smokers and CODP patients. There is also evidence that epigenetic ageing is associated with early decline in lung function and with COPD and may serve as a biomarker [37,46,47]. However, the different and sometimes contradictory results of the different studies are most likely due to a significant lack of harmonization of the studies. It is therefore difficult to deduce which epigenetic changes are actually relevant in that they are characteristic of COPD and may also be causally related to the progression of the disease. For example, studies use different methods to measure DNA methylation, and study populations vary significantly. Moreover, confounding factors such as BMI or smoking status are often not sufficiently considered, and methodologies are inadequately described [12]. Therefore, well-designed studies that accurately define cohorts and strictly account for confounding factors are needed to define epigenetic biomarkers of lung-function trajectories leading to COPD and thus to support precise treatment strategies [14].

## 3. Asthma

Allergic bronchial asthma is characterized by episodes of acute broncho-obstruction, shortness of breath, productive cough, wheezing, and the development of airway hyperresponsiveness (AHR). These symptoms arise in response to a chronic allergic inflammation of the airways and its interaction with the structural cells of the airways. Consequently, disentangling the processes and mechanisms underlying the formation of this inflammatory reaction still remains a major area of asthma research. 

For decades, it has been accepted that this allergic airway inflammation is centrally orchestrated and maintained by parts of the adaptive immune system. In particular, allergen-specific T helper 2 (TH2) cells and IgE-producing B cells, which are not only capable of differentiating between “self” and “non-self”, but also create an immunological memory of “non-self”, have been the focus of research aimed at answering the question of why the immune system reacts with an anti-parasitic response upon inhalation of usually harmless environmental antigens originating from, e.g., grass, birch, or hazelnut pollens or house dust mites (HDM). By releasing a characteristic array of cytokines such as interleukins (IL-) 4, -5, and -13, as well as granulocyte-macrophage colony-stimulating factor (GM-CSF), TH2 cells direct recruitment and maturation of eosinophil precursors, initiate class switching in B cells to produce IgE, and enhance mucus production in airway epithelia and submucosal glands. Consequently, it is undoubted that the adaptive immune system is central to asthma pathogenesis. However, the view that its two characteristic abilities—namely the differentiation between “self” and “non-self” and the creation of a memory of “non-self”—are exclusive attributes of the adaptive immune system had to be adapted: the discovery of toll-like receptors 30 years ago clearly demonstrated that innate immune cells such as macrophages or DCs are indeed able to recognize and to react to multiple microorganisms and thus are somehow capable of differentiating between “self” and “non-self”. Ten years ago, functions related to immunological memory were also attributed to cells of the innate immune system and were termed “trained immunity” [48]. Of course, the mechanisms involved in the creation of a memory of “non-self” are fundamentally different: while the adaptive immune memory utilizes genomic rearrangements that remain over many years, for example, in memory T or B cells, trained immunity is arranged by several regulatory epigenetic mechanisms such as DNA methylation, histone acetylation, and transcription of non-coding (nc) RNAs; these changes last for up to several months and can be reversed. Several studies found differences in DNA methylation, ncRNA expression [49,50,51], and histone acetylation [52,53] between asthma patients and healthy individuals; however, most studies on epigenetics in asthma patients have concentrated on DNA methylation [54], while other modifications have been studied considerably less frequently. Differences in DNA methylation have been associated with a number of immunological parameters such as eosinophil counts, IgE level, and expression levels of TH2-type cytokines such as IL-4 or IL-13 [55,56,57]. In particular, expression of these two cytokines and, subsequently, differentiation or polarization of TH2 cells, have been shown, both in vitro and in mouse models, to be regulated by miRNAs, which therefore affect the development of experimental asthma [58,59].

The impact of pulmonary exposure to environmental factors, including microorganisms and/or their products, has been discussed and investigated for decades, and it currently appears that their impact during critical periods of development (in utero or in early childhood) could be decisive. Thus, the hygiene hypothesis states that contact with particular microorganisms in early childhood can result in protection against the development of allergic diseases like asthma. Stein et al. found evidence for such an effect by comparing Amish and Hutterite farm children in the United States. These religious and agricultural populations have similar genetic ancestries and lifestyles but differ in farming practices and thus in exposure to microbes. Hence, median endotoxin levels in dust were 6.8 times higher in Amish households than in Hutterite households, whereas the prevalences of asthma and allergic sensitization were 4 and 6 times lower, respectively, among the Amish. This difference could be attributed to innate immunity [60]. Whether this effect is indeed mediated through trained immunity remains to be investigated; however, a study by DeVries et al. suggested that a mother’s prenatal immune status impacts the development of asthma in her child via epigenetic alterations and trained innate immunity at birth and that these variables could be affected by pathologic microbial colonization of the upper respiratory tract [61]. Further studies support the view that other factors, such as exposure to smoke or traffic-related airborne particles, could also lead to epigenetic modifications that might predispose individuals towards the development of asthma [62,63,64]. 

Nieto et al. provided evidence that trained immunity indeed impacts asthma pathogenesis. In this placebo-controlled clinical trial with 120 preschool children at risk of infection-associated recurrent wheeze, the authors demonstrated that a six-month oral treatment with a mixture of six heat-inactivated common bacterial pathogens significantly lowered wheezing attacks and reduced symptoms as well as medication scores. Remarkably, the effects lasted for up to six months after the end of the treatment [65].

Actually, it appears that not only the time point of exposure, but also the pattern or combination of microbes, decides whether trained immunity increases or reduces an individual’s susceptibility to asthma development. Hence, recurrent infection with respiratory viruses (e.g., respiratory syncytial virus or rhinovirus) or exposure to several bacteria (e.g., *M. catarrhalis* or *H. influenzae*) increases the risk for asthma development in later life [66]. Thus, on the one hand, Machiels et al. showed for the first time that an infection-induced protective effect against the development of asthma arises as a result of long-term programming of innate immune cells. Here, pulmonary infection with murid herpesvirus 4 (MuHV-4) protected mice from the induction of house dust mite-induced experimental allergic asthma (HDM-EAA) 30 days later. Specifically, this study demonstrated that in MuHV-4 infected mice, resident alveolar macrophages were replaced by bone marrow-derived (BMD) monocytes exhibiting lower expression of co-stimulatory molecules and increased secretion of IL-10, which in turn blocked the ability of dendritic cells (DC) to induce TH2 cell responses to HDM. These monocytes maintained their regulatory capacity and locally persisted in the lungs of infected mice for more than four weeks, leading to the conclusion that the previous MuHV-4 infection led to trained immunity [66]. 

On the other hand, two studies demonstrated the opposite, namely, that trained immunity propels the development of experimental asthma in mice. Based on the observation that asthma prevalence increased in Taiwan after various epidemics of enterovirus 71 (EV-A71), Chen et al. investigated the effect of neonatal EV-A71 infection on the development of HDM-EAA in mice. Indeed, intra-peritoneal EV-A71 infection at an early age of 14 days led to innate immune training of macrophages, which released more CCL17, IL-6, and tumor necrosis factor (TNF). This proinflammatory M2 status persisted over time and could be blocked by 2-deoxy-D-glucose (2-DG), which blocks glycolysis in cells engaged in trained immunity. Consequently, infected mice displayed increased titers of HDM-specific IgE and disease pathology when they were subjected to induction of HDM-EAA three weeks later. This effect could be reproduced in naïve mice that received adoptive transfer of EV-A71-trained BMD macrophages prior to induction of HDM-EAA, indicating that EV-A71-trained macrophages are able to favour the development of allergic asthma in later life [67,68]. In line with these findings, Rajput et al. observed that early-life infection alters the response to subsequent infection and produces an exaggerated asthma-like phenotype in mice. They additionally demonstrated that this effect is due to immune training of type 2 innate lymphoid tissue cells (ILC2) triggering increased AHR, mucus production, and expression of T2-type cytokines in the lung [69]. Typically, ILC2 activated by the epithelium-derived alarmin IL-33 start to proliferate and to release cytokines like IL-5 and IL-13. Therefore, they have been suggested as central players in those processes that lead to allergic sensitization through the airways. Steer et al. showed in mice that this activation by IL-33 can also be memorized by ILC2 cells if it happens in early childhood and that these trained ILC2 that become long-lasting residents in the lung can facilitate the development of experimental asthma later in life [70]. Subsequently, Verma et al. found that NFκB1 is a critical regulator of such immune memory [71]. Eljaszewicz et al. provided the first evidence that training of ILCs is also implicated in the beneficial effects of allergen-specific immunotherapy in asthma patients [72]. 

Furthermore, Lechner et al. also investigated the role of macrophages in the pathogenesis of allergic bronchial asthma, since they observed that monocyte-derived macrophages of HDM-allergic asthma patients, as well as BMD macrophages from mice with HDM-EAA, revealed persistently upregulated expression of type 2 (T2)-inflammatory chemokines. They further found that macrophages from these mice initially displayed a T2-imprint; however, this phenotype shifted towards a classical proinflammatory phenotype characterized by expression of IL-6 and CCL17 during induction of HDM-EAA. This suggests that HDM, which naturally contains several microbial compounds, including LPS, and HDM-induced inflammation elicit the activated M2 macrophage phenotype [73]. Quite recently, Han et al. also described differences in mRNA modifications, namely N^6^-methyladenosine (m^6^A) levels, in children with allergic asthma. Based on the finding of low-level expression of the m^6^A methyltransferase METTL3 in monocyte-derived macrophages from these patients, they demonstrated that METTL3 knock-out in myeloid cells elevates TH2 cell responses and consequently aggravates allergic airway inflammation in mice by facilitating M2 macrophage activation [74]. 

Together, these studies indicate that immune training, which can be interpreted as an adaption of innate immune cells to make responses to future infections stronger, can unintentionally promote unwanted inflammation through the enhanced release of proinflammatory cytokines and thus increase the risk for the development of chronic inflammatory diseases such as asthma. 

These initial works demonstrate that trained immunity—and thus epigenetics in innate immune cells—could play an important role in the development of allergic asthma and could even be decisive right at the beginning: they clearly show that innate immune cells such as macrophages establish and maintain a memory of contacts with bacteria or viruses and that this memory increases or decreases the probability of developing allergic sensitization and subsequent airway inflammation. In summary, two fundamental conclusions can be drawn from these findings: firstly, this memory is not restricted to the compartment where contact with the bacteria or viruses occurred, so that, e.g., immune training that was established in the gut or peritoneum has the potential to affect immune responses in the lung. Secondly, these studies demonstrate that trained immunity is not a fixed state, but can be reprogramed, either by environmental factors that may lead to progression of the disease or—and this option is now open—by a targeted approach to direct trained immunity in the opposite direction to create a new therapeutic strategy.

All these studies focused on the contribution of the epigenetic training of innate immune cells like macrophages to asthma pathogenesis. This, however, leaves open the question of whether other cells capable of reacting to microbial compounds could create an innate immune memory and affect disease development. This is especially relevant for airway epithelial cells (AECs), which are central to asthma pathogenesis, and a few initial studies already identified different DNA methylation signatures and some differences in histone acetylation in AECs from patients with asthma [75,76,77]. 

Undisputedly, the main task of AECs is to constitute the barrier between the body and its environment, and thus, the cells are not able to migrate through vessels and tissues like classic immune cells do. Nevertheless, AECs express a broad array of pattern-recognition receptors and are often among the first cells to initiate acute inflammation in reaction to microbe contact. They thus contribute to the defence of our body in an essential way. Additionally, as a central part of the epithelial-mesenchymal trophic unit (EMTU), AECs are centrally involved in airway remodelling; they represent the target cells for most of those respiratory viruses that have been implemented in the initiation and exacerbation of asthma, and upon epithelial damage, they release factors favouring T2-type immune reactions such as IL-25, IL-33, and thymic stromal lymphopoietin (TSLP) [78]. These characteristics make AECs central to the pathogenesis of asthma; however, one must realize that most studies on epigenetics in AECs carried out in the context of asthma did not use AECs from the lung, but rather focused on nasal epithelial cells (NECs). Using parallel single-cell RNA sequencing of NECs from patients with chronic rhinosinusitis, Ordovas-Montanes et al. provided the first hint of evidence for innate training of AECs. Here, they demonstrated that basal cells retain an intrinsic memory of exposure to TH2-type cytokines when cultured ex vivo, and they concluded that these potentially contribute to disease persistence by serving as repositories for a kind of an “allergic memory” [79]. Another study by Bigot et al. demonstrated in vitro that respiratory epithelial cells establish a memory of homologous and heterologous exposure to different bacteria and bacterial compounds affecting the subsequent response (e.g., the release of proinflammatory cytokines like IL-1 and IL-8) to the next stimulation. Remarkably, the combination of exposures decided the extent of this subsequent response—an outcome that is in line with those of the above-mentioned studies in murine macrophages [80]. 

In conclusion, the investigation of the role of epigenetics and especially trained immunity in the pathogenesis of asthma is still in its early stages, but it could contribute to our understanding of why different people are at differential risk of developing asthma or frequently experiencing complications of asthma. 

## 4. IPF

IPF is a fatal lung disease of unknown aetiology for which there is no satisfactory treatment. In IPF, the formation of scar tissue in the lungs is caused by the overactivation of fibroblasts, an increase in the deposition of extracellular matrix, and the loss of pneumocytes [3]. Pneumocyte injury and dysfunction is thought to be an early initiating event in IPF [81]. Risk factors for IPF include age, genetic predisposition, autoimmune diseases, and gastroesophageal reflux, but also environmental factors such as air pollution and smoking [82].

Only a few studies have examined epigenetic changes in samples from patients with IPF. In one of the first studies, Sanders et al. analysed lung tissue from 12 IPF patients and 7 controls [83]. In IPF lung tissue, 460 genes exhibited decreased methylation levels, while 464 genes demonstrated elevated methylation levels. There were also 16 genes whose mRNA expression showed an association with DNA methylation [83]. In another study involving lung tissue from 12 IPF patients and 10 controls, 625 CpG islands were found to be differentially methylated. The authors suggest that the genes associated with the differentially methylated CpG islands are involved in the regulation of apoptosis, morphogenesis, and cellular biosynthetic processes [84]. Genome-wide differences in DNA methylation have also been found in cultured fibroblasts from IPF patients compared to cultured fibroblasts from healthy controls. However, these studies analysed very few isolates (six or eight IPF patients and three or four controls) [85,86]. Hanmandlu et al. performed an assay of transposase-accessible chromatin sequencing (ATAC-Seq) using cultured fibroblasts from the lungs of three IPF patients and three healthy controls [87]. Several motifs were identified that were enriched in IPF fibroblasts, including binding motifs for *TWIST1* and *FOXA1*. The expression of 93 genes could be annotated to differentially accessible regions. Increased expression of *TWIST1* and *FOXA1* was also found in isolated lung tissue from other IPF patients [87]. A study analysing macrophages from BAL fluid (30 IPF patients and 14 healthy donors) identified epigenetic heterogeneity as a key feature of IPF. The differentially methylated positions and regions identified included genes involved in lipid and glucose metabolism. DNA methylation status was also associated with disease severity [88]. 

There is strong evidence that epigenetic modifications regulate the expression of genes associated with IPF. rs35705950 is a MUC5B risk allele for IPF. Studies suggest that increased methylation and chromatin accessibility of an enhancer region encompassing the rs35705950 variant is associated with aberrant MUC5B expression in IPF [89,90,91]. Valenzi et al. identified TWIST1 as a highly enriched regulator of myofibroblast activity in IPF in an in-depth study using multiomic single-cell analysis, including ATAC-Seq, on explanted lungs. TWIST1 was significantly enriched in the open chromatin of myofibroblasts from IPF patients, and overexpression of *Twist1* in fibroblasts resulted in increased expression of collagen I and α-SMA [92].

The extent to which epigenetic changes contribute to the pathogenesis of IPF remains uncertain [93]. However, it is becoming evident that the hypermethylation and accessibility of specific genes, such as *MUC5B* or *TWIST1*, in lung cells exert a pivotal influence on the progression of IPF and contribute to the loss of lung function. The extent to which epigenetic alterations induced by environmental factors such as smoking or by diseases, including autoimmune disorders and gastroesophageal reflux, are causally related to IPF remains uncertain.

## 5. Cystic Fibrosis

Cystic fibrosis (CF) results from mutations in the gene encoding the cystic fibrosis transmembrane conductance regulator (CFTR). The CFTR protein is an ion channel in the cell membrane that regulates the transport of chloride ions. Mutations in the CFTR gene lead to impaired chloride transport, resulting in the production of thick mucus that causes recurrent infections, inflammation and, ultimately, lung damage [94]. To date, there have been few studies on the role of epigenetics in CF. Analysis of BAL cells (four CF patients, four healthy controls) revealed 109 differentially methylated CpGs (51 hypermethylated, 58 hypomethylated) [95]. Magalhaes et al. analysed nasal epithelial cells obtained from the inferior turbinate of 32 CF patients and 16 controls. Here, 1267 differentially methylated CpGs (303 hypermethylated, 318 hypomethylated) were associated with 638 genes. Furthermore, DNA methylation levels differed between mild and severe CF patients and were correlated with lung function (FEV1%) at 50 CpG sites [96]. Future studies must show the extent to which epigenetic changes resulting from the disease further promote susceptibility to acute and chronic infections, are involved in disease-relevant processes such as the formation of reactive oxygen species, and correlate with a loss of lung function [97]. Questions arise as to the extent to which CF-relevant genes are differentially methylated and whether epigenome editing can be employed as a therapeutic strategy [98,99]. At least preclinical cell culture studies suggest that the expression of CFTR and macrophage function can be enhanced by therapeutic intervention, thereby improving the efficacy of CFTR modulators [99,100].

## 6. Conclusions

**Figure 2:**
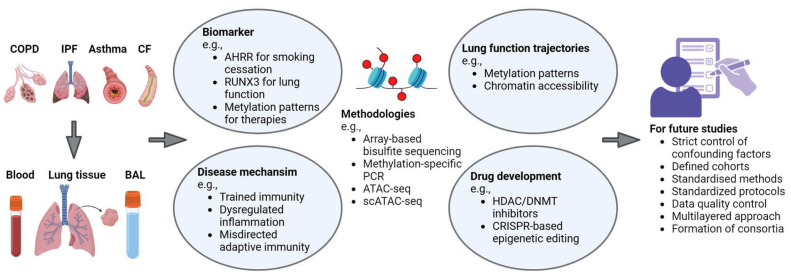
Numerous studies have analysed blood cells, lung tissue, and bronchoalveolar lavage (BAL) cells from patients with chronic lung disease to identify epigenetic changes. These studies are providing insights into potential biomarkers and disease mechanisms that could contribute to the development of new therapeutic strategies. However, the considerable heterogeneity among studies, coupled with shortcomings such as poorly defined confounders and small sample sizes, highlight areas that should be addressed in future research. (Created in BioRender. Beissswenger, C. (2025) Created in https://BioRender.com/o80m047 (accessed 10 February 2025)).

Numerous studies indicate that epigenetic modifications play a crucial role in the development and progression of chronic lung diseases by regulating genes and influencing disease-relevant cellular processes and signalling pathways. Environmental factors such as poverty, smoking, and air pollution, as well as conditions like gastroesophageal reflux, may be associated with epigenetic changes long before a disease is diagnosed. Thus, epigenetic signatures in patient samples (e.g., blood cells) may serve as biomarkers that can be used to monitor lung function trajectories and identify young patients at risk for premature loss of lung function or diseases such as IPF. Epigenetic signatures may also serve directly as biomarkers, as the demethylation of AHRR does for smoking status. In addition, epigenetic analyses contribute to a better understanding of disease mechanisms, including cytokine dysregulation, misdirected adaptive immune responses, and the role of trained immunity. These analyses hold immense potential to advance our knowledge of chronic lung diseases and drive the development of innovative treatments and drugs (e.g., HDAC and DNMT inhibitors, CRISPR-based epigenetic editing) (Figure 2).

Although numerous epigenetic studies have been conducted on diseases such as COPD, these efforts have been highly heterogeneous, with the majority focusing exclusively on DNA methylation. As a result, efforts to derive reliable biomarkers and use the data to understand disease mechanisms have been challenging, if not impossible. While each study offers valuable insights individually, the lack of consistency and scope limits their collective utility. For future studies, strict control of confounding factors—such as smoking history, comorbidities, and environmental exposures—will be essential. Cohorts should be well-defined, with a focus on specific disease subtypes to reduce heterogeneity. Standardized methods and protocols for data collection and analysis are crucial to enhance comparability across studies, while rigorous quality control for data will ensure reliability and reproducibility. Additionally, a multilayered approach that integrates various epigenetic modifications (e.g., DNA methylation and chromatin accessibility) with gene expression could provide a more comprehensive understanding of disease mechanisms. Furthermore, the formation of international consortia would enable large-scale, collaborative efforts to overcome these challenges and generate more robust, clinically relevant findings. 

Epigenetic analysis is not yet a routine part of daily clinical practice. This raises the question of the extent to which epigenetic analysis can be integrated into clinical practice for the benefit of patients in the future. Currently, epigenetic testing is primarily utilized in clinical research and specialized centres, but with ongoing advancements, it is expected to become an integral part of diagnosis, treatment selection, and disease monitoring in routine medical practice. It is crucial for basic researchers to collaborate closely with clinicians to design studies that align with clinical needs (e.g., up-to-date treatment strategies), ensuring that epigenetic tests—such as those predicting therapy responses—are both clinically relevant and cost-effective. By addressing these challenges, future epigenetic studies could unlock critical insights that not only improve our understanding of chronic lung diseases, but also pave the way for early detection, personalized treatments, and novel therapeutic strategies. 

## Data Availability

Not applicable.
